# A Rare Cause of Pulmonary Hypertension in a 4-Year-Old Toddler: Association of Cor Triatriatum Sinister and Pulmonary Arteriovenous Malformation

**DOI:** 10.1155/2020/8825215

**Published:** 2020-11-11

**Authors:** Can Yilmaz Yozgat, Erkan Cakir, Hakan Yazan, Hafize Otcu Temur, Kahraman Yakut, Yilmaz Yozgat

**Affiliations:** ^1^Faculty of Medicine, Bezmialem Vakif University, Istanbul, Turkey; ^2^Department of Pediatric Pulmonology, Bezmialem Vakif University, Istanbul, Turkey; ^3^Department of Radiology, Bezmialem Vakif University, Istanbul, Turkey; ^4^Department of Pediatric Cardiology, Bezmialem Vakif University, Istanbul, Turkey

## Abstract

Cor triatriatum sinister is a rare congenital cardiac anomaly. The anomaly is caused by a fibromuscular membrane that divides the left atrium into two cavities. This membrane can lead to the obstruction of left atrial flow and also create pulmonary venous hypertension. Pulmonary arteriovenous malformation (PAVM) is notorious for its aberrant blood flow between the pulmonary arteries and veins. Herein, we report a case of a 4-year-old toddler who had a unique form of pulmonary hypertension presenting with cor triatriatum sinister and diffuse PAVM. After the surgical treatment of cor triatriatum sinister, both pulmonary arteriovenous malformation and pulmonary hypertension disappeared.

## 1. Introduction

Cor triatriatum sinister is a rare congenital cardiac anomaly. The anomaly is caused by a fibromuscular membrane that divides the left atrium into two cavities. This membrane can lead to the obstruction of left atrial flow and also create pulmonary venous hypertension [1]. Pulmonary arteriovenous malformation (PAVM) is notorious for its aberrant blood flow between the pulmonary arteries and veins. PAVM can be either congenital or acquired. The acquired causes PAVM might include hepatic cirrhosis, post bidirectional Glenn shunt, and congenital heart disease as mitral stenosis [2, 3]. Herein, we report a case of a 4-year-old toddler who had a unique form of pulmonary hypertension presenting with cor triatriatum sinister and pulmonary arteriovenous malformation. To the best of our knowledge, this is the first case reported in the literature. After the surgical treatment of cor triatriatum sinister, both pulmonary arteriovenous malformation and pulmonary hypertension resolved.

## 2. Case Report

**Figure 1:**
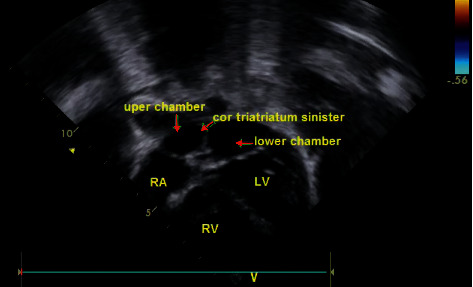
TTE demonstrating the left atrium expansion which has a fibromuscular membrane which divides the left atrium into two chambers.

**Figure 2:**
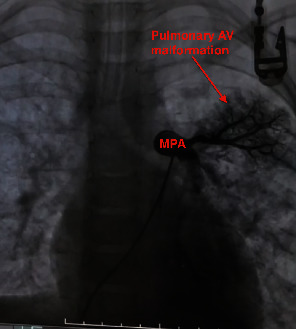
TEE revealing cor triatriatum having two fenestrations: one with a width of 2 mm and the other with 3 mm diameter.

A male, 4-year-old, was admitted to our hospital with persistent respiratory symptoms for three consecutive years. On his physical examinations during three consecutive years, the patient's SpO2 was 98% in the first checkup; after that, his SpO2 gradually reduced to 90% in room air, and clubbing of the fingers has gradually occurred during the same time. His vital findings of blood pressure, heart rate, and respiratory rate were normal, both lungs were equally ventilated, and mild wheezing existed in the lungs. Chest X-ray pointed out bilateral infiltrations in the lungs; a prominent hilum was discovered. There was no evidence suggestive of a focal air space disease. Transthoracic echocardiogram (TTE) demonstrated that the left atrium was expanded and had the fibromuscular membrane, which divided the left atrium into two chambers (Figure 1). The interatrial septum was intact. RV systolic pressure was measured to be 50 mmHg on echocardiography. Transesophageal echocardiography (TEE) was performed to have a better view of the membrane. TEE revealed cor triatriatum had two fenestrations: one with a width of 2 mm and the other with 3 mm diameter. A feneration had a maximum pressure gradient by continuous-wave Doppler of 20 mmHg (Figure 2). To be able to evaluate the patient's pulmonary vascular bed, a heart catheterization was performed. Right atrial pressure was a mean of 6 mmHg. The pulmonary artery pressure was 60/24, with a mean of 38 mmHg. Right ventricular pressure was 60/6 mmHg.

**Figure 3:**
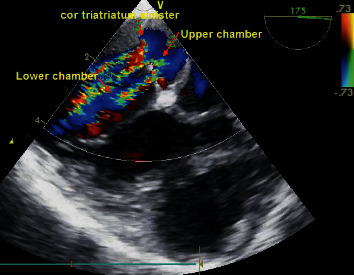
Selective pulmonary angiography showing AV malformation in the upper part of the left lung.

Furthermore, a selective pulmonary angiography was performed in addition to cardiac catheterization to evaluate the patient's pulmonary venous drainage and the pulmonary bed. The results were extremely intriguing. The angiography showed the late opacification of drainage veins connecting to the other pulmonary vein from the different segments of the left lung (Figure 3). The angiography disclosed the mimicking of pulmonary venous stenosis/obstruction due to the existence of restrictive cor triatriatum sinister. There was a diffuse PAVM in the upper part of the left lung as well. Contrast echocardiology (CE) was performed with a manual injection of 10 mL of agitated saline. Pulmonary AVM diagnosis was confirmed by the appearance of bubbles in the left atrium after more than three cardiac cycles after the first bubble was detected in the right atrium. Also, the patient's thorax CT was evaluated as PAVM after the cardiac catheterization.

Also, the patient continued to have pulmonary venous hypertension and cor triatriatum sinister with restrictive fenestration. The patient's dilemma was discussed in a council. The council was composed of pediatric cardiologists, pediatric cardiovascular surgeons, and thoracic surgeons. The final decision was to perform a surgical membrane resection to follow PAVM. After the surgery, on his first postoperative follow-up time, pulmonary artery pressure was reasonable and PAVM completely resolved.

## 3. Discussion

Clinical presentation of cor triatrium sinister depends on the diameter of fenestration. Routine clinical symptoms include deteriorating respiratory system disorders such as dyspnea, orthopnoea, and hemoptysis (1). TTE examination is the best noninvasive method to diagnose cor triatriatum sinister and approximately measure the pulmonary artery pressure [3]. It has been reported that severe obstruction is indicated by maximum Doppler velocity, which has to be higher than 2 m/s [4]. In this case, we suspected the existence of cor triatrium sinister in a TTE examination. TEE revealed cor triatriatum had two different fenestration. The maximum gradient was 20 mmHg, which was measured by CW Doppler. When the membrane constituted a restriction of the left atrial flow, it might mimic the characteristics of mitral stenosis, which causes a subsequent rise in proximal left atrial pressure, pulmonary congestion, and pulmonary venous hypertension [4].

PAVM can be either congenital or acquired. 80% of all cases are hereditary. Most of the patients with PAVM have a higher chance of carrying Osler–Weber–Render disease or hereditary hemorrhagic telangiectasia (HHT) [2, 5]. Our case has not shown any signs of hereditary disease. Secondary or acquired PAVM has been rarely reported in the literature. Causes of secondary PAVM include chest trauma, thoracic surgery, long-standing hepatic cirrhosis, metastatic carcinoma, and mitral stenosis [5, 6]. The best way to detect the existence of PAVM and pulmonary hypertension is to perform cardiac catheterization and selective angiography [3, 4]. In this case, the existence of PAVM was accidentally found when the cardiac catheterization had been performed to evaluate pulmonary hypertension. The results were fascinating. There was an AV malformation in the upper part of the left lung, and pulmonary artery pressure was extremely high. Surgical resection is the best treatment option to fix cor triatriatum and PAVM [3, 4]. For this case, the membrane was resected; however, the patient continued to have pulmonary arteriovenous malformation. After the surgery, the toddler did exceptionally well throughout the first six months. The pathophysiological formation of acquired PAVM patients is unclear, but our theory is that since the obstruction is removed after surgery, venous engorgement is relieved. As a theory, the long-standing pulmonary venous engorgement could cause PAVM because of increased hydrostatic pressure on venous vasculature. Another angiography was performed in the first year of follow-up. Angiography indicated that PAVM completely vanished, and pulmonary artery pressure was normal. To the best of our knowledge, this is the first report of co-occurrence of cor triatriatum sinister and PAVM. If detection of PAVM and pulmonary hypertension is made, a thorough investigation should have been done with TEE to search restrictive cor triatrium sinister.

## Data Availability

The data supporting the current study are available from the corresponding author upon reasonable request.

## References

[1] Rodefeld M. D., Brown J. W., Heimansohn D. A. (1990). Cor triatriatum: clinical presentation and surgical results in 12 patients. *The Annals of Thoracic Surgery*.

[2] King I., Downey G. H. (2002). Pulmonary arteriovenous malformation. *Postgraduate Medical Journal*.

[3] Meek M., Meek J., Beheshti M. (2011). Management of pulmonary arteriovenous malformations. *Seminars in Interventional Radiology*.

[4] Slight R. D., Nzewi O. C., Buell R., Mankad P. S. (2005). Cor-triatriatum sinister presenting in the adult as mitral stenosis: an analysis of factors which may be relevant in late presentation. *Heart, Lung and Circulation*.

[5] Houston A., Hillis S., Lilley S., Richens T., Swan L. (1998). Echocardiography in adult congenital heart disease. *Heart*.

[6] Prager R. L., Laws K. H., Bender H. W. (1983). Arteriovenous fistula of the lung. *The Annals of Thoracic Surgery*.

